# Thyroid papillary carcinoma associated to Hashimoto's thyroiditis

**DOI:** 10.5935/1808-8694.20120037

**Published:** 2015-10-20

**Authors:** Luiz Alexandre Albuquerque Freixo Campos, Sílvia Miguéis Picado, André Vicente Guimarães, Daniel Araki Ribeiro, Rogério Aparecido Dedivitis

**Affiliations:** a General Practice Resident Physician at Hospital Ana Costa, Santos; b Resident Physician at Hospital Regional do Vale do Paraíba, Taubaté; c PhD - Department of Surgery - Medical School of the University of São Paulo. Assistant Physician - Head and Neck Surgery Department at Hospital Ana Costa, Santos; d Adjunct Professor and Researcher - Department of Biosciences - Pathology Program - Federal University of São Paulo; e MD. Senior Associate Professor - Fundaçã o Lusíada UNILUS. Department of Head and Neck Surgery - Ana Clara Hospital - Santos

**Keywords:** carcinoma, papillary, thyroid neoplasms, thyroiditis, autoimmune

## Abstract

There is controversy in the literature regarding the association between papillary thyroid carcinoma (PTC) and Hashimoto's thyroiditis (HT) and as to what would be the etiological relationship between them.

**Objective:**

To establish the proportion of cases among patients with TH and CPT, correlating it with histomorphological aspects.

**Method:**

A retrospective study of patients undergoing partial or total thyroidectomy for PTC between 2007 and 2009, a total of 41 cases.

**Results:**

Regarding the association of HT and CPT, we found 11 cases (26.8%), all females, but without statistical significance. The mean age was 44.9 years among the patients with coexistent TH and CPT, whereas it was 49.1 years without that association. The average size of tumors in those without TH was 20.53 mm and for those with TH it was 12.72 mm (*p* = 0.4). Regarding pathology staging, the ratiosbetween those with and those without TH were kept in T1a, T1b and T3. In T2, there were no cases of coexistence of HT and PTC.

**Conclusion:**

There is a rate of 26.8% of patients with association between TH and CPT, but without differences in relation to tumor size.

## INTRODUCTION

Papillary thyroid carcinoma (PTC) is the most prevalent and the one with the best prognosis among all malignant thyroid neoplasias^1^, ^2^. Hashimoto's thyroiditis (HT) is a chronic lymphocytic thyroiditis, and the most common cause of hypothyroidism in areas with proper amounts of iodine^3^. In 1955, Dailey et al.^4^ proposed, for the first time, the association between PTC and HT, also suggested by numerous other authors^5^, ^6^, ^7^, ^8^, ^9^, ^10^, ^11^.

However, there are authors who disagree from such association^12^, ^13^, ^14^ in studies which were not clear as to the association, after following up patients with HT. One of the bases for discussion is the etiology, and it is not clear whether thyroiditis predisposes to cancer or if it is later induced by the neoplasia^5^.

There are studies which show the expression of RET/PTC-1 and RET/PTC-3 oncogenes in patients with HT, confirmed by phenotyping^15^, ^16^, which could prove HT as a pre-neoplastic lesion. There was also a study which showed the concurrent p63 expression, a p53-homologous protein which regulate squamous cells in HT-suggestive cells without concurrent neoplasia and in typical PTC cells; present in high rates (around 80%) in these two groups and without significant expression in other thyroid involvements^17^.

Our paper aims at studying the possible significant association between these two entities and whether or not there are differences in the PTC presentation in patients with HT and those without. The goal is to find the incidence of HT cases in patients with PTC, correlating histomorphometric aspects.

## METHOD

The present study was approved by the Ethics in Research Committee of the institution where it was carried out, under protocol # 013/2011.

We carried out a retrospective study based on data collected from the files of the hospital where the study was carried out, as well as from the charts of patients submitted to total or partial thyroidectomies, because of PTC in histopathology specimens, between the years of 2007 and 2009, making up a total of 41 cases. We consecutively selected all the PTC cases, regardless of age or gender, with or without HT. We also investigated the incidence of Hashimoto's thyroiditis in the remaining cases submitted to partial or total thyroidectomies for other reasons. We took off the study those patients with incomplete data in their charts.

The study was based on data collected from the patients' charts, such as age, gender, tumor size and pathology staging after surgery.

The statistical analysis was carried out after preparing the data base. We then analyzed all the variables, investigating the statistical significance among the data, using the Fisher's exact test.

## RESULTS

In the time frame between 2007 and 2009, there were 315 thyroidectomies, 41 due to thyroid papillary carcinoma and 274 for other reasons. Of these 274 procedures, 45 were partial thyroidectomies because of nodular goiter and, in none of them the pathologist found chronic thyroiditis. Among the cases submitted to total thyroidectomy (n = 229), chronic thyroiditis was seen in 12.25%; if we consider only those patients operated because of atoxic multinodular goiter in this group, the incidence of thyroiditis drops down to 6.65% (Table 1).

**Table 1:** Thyroiditis in patients submitted to total thyroidectomy in the same period, taking off the cases of papillary carcinoma.

	Thyroiditis present	Without thyroiditis
Toxic diffuse goiter	9	0
Hashimoto's thyroiditis	3	0
Other cancers	0	6
Atoxic multinodular goiter	13	198
Total	25	204

Thus, there were 41 total or partial thyroidectomies due to papillary carcinoma of the thyroid. Of these, 11 had concurrent HT (26.8%), while 30 did not have it (72.1%) - Table 2.

**Table 2:** Ratios between genders and the coexistence of PTC and HT.

	PTC without HT (n/%)		PTC with HT (n/%)		total
Males	7	100	0	0	7
Females	23	67.6	11	32.5	34
Total	30		11		41

Thus, we found 11 cases of HT in 41 patients with papillary carcinoma (26.8%) and 13 cases of HT in 198 patients with multinodular goiter (6.65%), with statistical significance considering the Fisher's Exact Test *(p=* 0.005823).

Considering the demographic data from the 41 patients, 34 (82.93%) were females, while seven (17.07%) were males.

Concerning the HT-PTC association, there were 11 cases, all females, but without statistical significance. The mean age of the PTC cases was 48 years. In those cases of PTC concurrent with HT, the mean age was 44.9 years and without the association this value was 49-1 years. The minimum age among the patients was 20 years and the maximum was 91.

The mean tumor size among those without thyroiditis was 20.53 mm and for those with thyroiditis it was 12.72 mm, nevertheless, without statistical significance - *p* = 0.4.

As to the pathology staging, the ratios between those with HT and those who did not have it were maintained in T1a, T1b and T3. In T2, there were no cases of coexistence between HT and PTC (Table 3).

**Table 3:** Rates of HT cases among the PTC stages.

	PTC without HT (n/%)		PTC with HT (n/%)		total
T1a	9	69.2	4	30.8	13
T1b	8	61.5	5	38.5	13
T2	4	100	0	-	4
T3	9	81.8	2	18.2	11
total	30	73.2	11	26.8	41

## DISCUSSION

HT was first described in 1912^18^ as an autoimmune disease, affecting 5% of the general population and involving the diffuse infiltration of the thyroid by lymphocytes, fibrosis and gland parenchyma atrophy.

Since 1955^4^, there are questions concerning the association between HT and PTC, with studies corroborating and studies disproving such association. This association has been reported by many authors, although showing different indices, probably due not only to different genetic factors and different environments, but also the numerous pathology definitions used in the many studies^7^.

A metanalysis showed that the HT incidence is 2.77 higher in patients with PTC when compared to patients with benign thyroid diseases. In addition, in patients with thyroid carcinoma, the association of HT is 1.99 times higher among those with PTC than in patients with other pathological types of thyroid cancer^11^. Indirectly, these findings my suggest a predisposition of HT patients to the development of PTC. In our series we found 26.8% of HT in 41 cases of papillary carcinoma and 6.65% in 198 patients with multinodular goiter (*p* = 0.005823). Notwithstanding, in the present series there were 45 patients submitted to less-than-total thyroidectomy because of nodular goiter, therefore a benign disease, and in none of them we found thyroiditis, which is explained by the fact that we carried out total thyroidectomy as a routine in cases of nodular disease associated with HT. Thus, these 45 cases could not be included in this statistical analysis.

Therefore, the RET/PTC, which is a RET rearrangement, a gene which codes a tyrosine-kinase receptor, is described as one of the mechanisms responsible for the association of HT and PTC^19^. This oncogene was found in the large majority of tissues with HT and without detectable PTC, which may show a progression to cancer from chronic thyroiditis^15^, ^20^. The presence of RET/PTC must not be synonymous with cancer, but rather a molecular mechanisms of carcinogenesis^21^.

Another mechanism described is the p63 expression, a tumor-suppression protein homologous to the p53, which is found in about 81% of the HT and PTC, and it is not found in normal thyroid tissues, Graves disease or other thyroid tumors^17^.

In the present study, 26.8% of the cases showed both HT and PTC, which may suggest a correlation, even without control groups for a proper statistical analysis; however, consistent with literature data, reporting that it may vary between 20% and 37.5%^3^, ^22^, ^23^.

The predominance of females in this study, in both groups of patients, namely those associated with HT and those without such association, is in agreement with the literature^3^, ^5^, ^8^, ^11^, an epidemiology matching PTC. It is known that thyroid autoimmune diseases are more commonly expressed in women when compared to men, and such trend is even more obvious in the post-menopausal period. However, our study did not yield statistically significant differences as far as gender is concerned, which may have been caused by the low number of men among the patients. The same can be inferred in relation to the mean age of those involved, there were no differences between the two groups, which is in agreement with the literature, with higher prevalence in individuals around 50 years of age^1^, ^2^, ^4^.

Among the different stages of the disease, there were no statistically significant differences between the presence and absence of concurrent HT, which is also in agreement with the literature^24^. Some authors suggest a less aggressive course of the disease when there is association between HT and PTC^3^, ^23^, with tendency towards a smaller tumor size, less lymph node involvement and longer sur-vival^25^. A population study did not find statistically significant difference based on the presence of HT, despite a tendency towards a better survival in cases of concurrent diseases^11^. The metanalysis of three studies, involving 2,552 patients, carried out in the same publication, suggested a better disease-free survival among patients with PTC associated with HT. similarly, the global survival was also higher in this group in four of five studies which analyzed this parameter. The precise reason for such difference is still unclear.

The ultimate confirmation of a causal association between HT and PTC would require a longitudinal prospective study of patients with HT. Right now, the best approach for patients with HT, especially those with the nodular variant of the disease, is a careful follow up, clinically as well as with cytopathological study of the nodules. The surgical approach should be the total thyroidectomy, since the cases of concurrent diseases are more frequently multicentric (93%) when compared to cases without the association (50%)^26^.

## CONCLUSION

There is a rate of 26.8% of patients with the HT-PTC association, however without statistical significance vis-à -vis tumor size. More studies are needed in order to assess the higher development of neoplasia in patients with HT and assess the possible factors which may trigger the carcinogenesis, thus contributing to prevention, early diagnosis and target treatment.
